# Diversity in growth patterns among strains of the lethal fungal pathogen *Batrachochytrium dendrobatidis* across extended thermal optima

**DOI:** 10.1007/s00442-017-3866-8

**Published:** 2017-04-19

**Authors:** Jamie Voyles, Leah R. Johnson, Jason Rohr, Rochelle Kelly, Carley Barron, Delaney Miller, Josh Minster, Erica Bree Rosenblum

**Affiliations:** 1 Department of Biology, University of Nevada-Reno, Reno, NV 87801 USA; 2 Department of Integrative Biology, University of South Florida, Tampa, FL 33620 USA; 3 Department of Statistics, Virginia Polytechnic Institute and State University, Blacksburg, VA 24061 USA; 4 Department of Biology, University of Washington, Seattle, WA 98195 USA; 5 Department of Biology, New Mexico Tech, 801 Leroy Place, Socorro, NM 87801 USA; 6 Department of Environmental Science, Policy and Management, University of California- Berkeley, Berkeley, CA 94720-3144 USA

**Keywords:** *Batrachochytrium dendrobatidis*, Amphibian chytridiomycosis, Disease ecology, Psychrophilic fungi, Temperature

## Abstract

The thermal sensitivities of organisms regulate a wide range of ecological interactions, including host–parasite dynamics. The effect of temperature on disease ecology can be remarkably complex in disease systems where the hosts are ectothermic and where thermal conditions constrain pathogen reproductive rates. Amphibian chytridiomycosis, caused by the pathogen *Batrachochytrium dendrobatidis* (*Bd*), is a lethal fungal disease that is influenced by temperature. However, recent temperature studies have produced contradictory findings, suggesting that our current understanding of thermal effects on *Bd* may be incomplete. We investigated how temperature affects three different *Bd* strains to evaluate diversity in thermal responses. We quantified growth across the entire thermal range of *Bd*, and beyond the known thermal limits (*T*_max_ and *T*_min_). Our results show that all *Bd* strains remained viable and grew following 24 h freeze (−12 °C) and heat shock (28 °C) treatments. Additionally, we found that two *Bd* strains had higher logistic growth rates (*r*) and carrying capacities (*K*) at the upper and lower extremities of the temperature range, and especially in low temperature conditions (2–3 °C). In contrast, a third strain exhibited relatively lower growth rates and carrying capacities at these same thermal extremes. Overall, our results suggest that there is considerable variation among *Bd* strains in thermal tolerance, and they establish a new thermal sensitivity profile for *Bd*. More generally, our findings point toward important questions concerning the mechanisms that dictate fungal thermal tolerances and temperature-dependent pathogenesis in other fungal disease systems.

**Electronic supplementary material:**

The online version of this article (doi:10.1007/s00442-017-3866-8) contains supplementary material, which is available to authorized users.

## Introduction

Temperature is one of the most critical abiotic factors regulating ecological processes. Owing to the rate-limiting influence of temperature on biochemical mechanisms, there is a rich and extensive literature on how the thermal sensitivities of organisms can shape their molecular and cellular biology, ecophysiology, behavior, abundance, and distribution (Johnston and Bennett 2008; Beveridge et al. 2010). In addition, there has been a great interest in establishing thermal sensitivity profiles for diverse organisms, and in investigating how thermal performance curves may have evolved (Johnston and Bennett 2008; Krenek et al. 2012). One area that has recently garnered considerable attention is how the field of thermal biology can be integrated with ecological and evolutionary studies of host–parasite interactions (Blanford and Thomas 1999; Krenek et al. 2012).

Current research is providing numerous examples of how thermal environments can shift disease dynamics and drive host–parasite coevolution (Blandford and Thomas 1999). Temperature can constrain both the ability of a host to defend itself, and a pathogen’s capacity to colonize and reproduce within a host. If the thermal sensitivity profiles of host and pathogen match, then the effects of temperature in a disease system may be relatively straightforward to understand. If, on the other hand, there is considerable diversity in thermal tolerances within and among host species, or among pathogen strains, then the effect of temperature can be remarkably complex and have important implications for the manifestation of disease. Therefore, investigating the intricacies of these interactions may provide insights into the temperature-regulated mechanisms of disease that have been difficult to resolve [e.g., disease systems that exhibit strong seasonal fluctuations (Dowell 2001; Koelle et al. 2005) or that are limited or exacerbated across altitudinal and latitudinal gradients (Guernier et al. 2004; Gilbert 2010)].

One disease that provides a compelling example of temperature-sensitive host–parasite interactions is amphibian chytridiomycosis (Berger et al. 2004; Raffel et al. 2010, 2013). Chytridiomycosis is caused by the fungal pathogen, *Batrachochytrium dendrobatidis* (hereafter *Bd*; Longcore et al. 1999). It has been suggested that *Bd* may have spread around the world relatively recently (although questions regarding the point of origin and the timing of spread are still debated; Rosenblum et al. 2013). In multiple geographic locations, *Bd* emergence in naïve host populations has caused precipitous declines in amphibian populations, including some extinctions (Skerratt et al. 2007; Schloegel et al. 2006). These declines have occurred in a wide variety of environments, including the deserts in temperate North America [e.g., *Lithobates* (*Rana*) *yavapaiensis* and *Lithobates* (*Rana*) *chiricahuensis* (Bradley et al. 2002)], high alpine, temperate regions in North America [e.g., *Rana muscosa* and *R. sierrae* (Briggs et al. 2010; Vredenburg et al. 2010)] and in tropical rainforests in Central America [e.g., *Craugastor punctariolis* (Ryan et al. 2008)] and Australia [e.g., *Taudactylus acutirostrostris* (Schloegel et al. 2006)].

Although there has been a general consensus among researchers that temperature plays an important role in this disease, the mechanisms that underpin the temperature effects on chytridiomycosis have not been fully resolved (Venesky et al. 2014). Amphibians rely on environmental heat sources to adjust their body temperatures (Richards-Zawacki 2010; Rowley and Alford 2013) and amphibian immune function is dependent on temperature (Raffel et al. 2006; Butler et al. 2013). In addition, laboratory studies suggest that *Bd* optimal growth and reproduction occur within a restricted thermal range of 4–25 **°**C (Piotrowski et al. 2004; Woodhams et al. 2008). However, field studies that have described chytridiomycosis outbreaks demonstrate that our understanding of temperature effects on *Bd* in the laboratory does not fully explain disease dynamics in the wild (Venesky et al. 2014).

Chytridiomycosis outbreaks in tropical regions have predominantly occurred at cooler, high-elevation sites and when temperatures are at seasonal lows (Berger et al. 2004; Woodhams and Alford 2005; Sapsford et al. 2013). In contrast, similar investigations in temperate regions have not detected seasonal, latitudinal or altitudinal patterns in infection or disease (Kriger and Hero 2008; Korfel and Hetherington 2014; Petersen et al. 2016). Because the role of temperature in chytridiomycosis in temperate regions is much less clear, some investigators have speculated that other environmental factors may outweigh temperature in determining disease outcomes (Knapp et al. 2011; Korfel and Hetherington 2014). Thus, while some studies suggest that the importance of temperature is unequivocal, other studies suggest that temperature plays virtually no role in chytridiomycosis outbreaks (Knapp et al. 2011; Korfel and Hetherington 2014). While these studies do not necessarily negate the importance of temperature, they nevertheless suggest that the effects of temperature are more nuanced than we initially appreciated (Venesky et al. 2014; Cohen et al. 2017).

One strong starting point for improving our understanding of thermal effects on *Bd* is to investigate its responses to temperature (1) decoupled from the confounding effects of host defenses, (2) among *Bd* strains from globally diverse sources, and (3) at the extremes of its thermal range.

We selected a panel of *Bd* strains from different geographic regions and evaluated the responses of these isolates in temperature shock treatments and across the entire known *Bd* temperature gradient (Piotrowski et al. 2004; Stevenson et al. 2013). We then used our empirical data to fit a mathematical model of *Bd* growth over our experimental temperature spectrum. This approach offered a controlled common garden experiment where we could track three *Bd* strains and their growth characteristics at different temperatures in real time. We hypothesized that the three *Bd* strains would exhibit variation in growth and viability in our experimental treatments.

## Methods

### *Bd* isolate selection

We selected three *Bd* isolates for our experiments from one of the largest global collections of *Bd* isolates available (see Rosenblum et al. 2013). Our primary aim was to choose representative strains from different clades within the *Bd* phylogeny. Our secondary aim was to use strains that originated from different thermal regions. Additionally, because laboratory maintenance practices can have profound effects on *Bd* growth patterns (Voyles et al. 2014), we selected *Bd* strains that were collected and cryo-archived with similar protocols (Boyle et al. 2003), and that had low passage histories.

CJB5-2 originated in the Sierra Nevada Mountains of California, and was isolated from *Rana muscosa*. Genomic data show that CJB5-2 is clearly nested within the global pandemic lineage (GPL; Rosenblum et al. 2013). Amphibian microhabitat temperatures in this region range from 4 to 28 **°**C (Knapp et al. 2011). We refer to CJB5-2 as “Temperate” throughout the paper. LFT originated in Reserva Biologica Serra do Japi in Tropical Brazil, and was isolated from *Hylodes ornatus.* We refer to LFT as “Tropical” throughout the paper. In this region, average annual temperatures range from 11 to 22 **°**C (Vieira et al. 2013). UM-142 was isolated from a bullfrog [*Lithobates* (*Rana*) *catesbeiana*] in an amphibian trade market in Michigan, USA. However, genetic analyses suggest that this strain may have originated in Latin America (although the location is unclear) and it was subsequently introduced to bullfrogs in the pet trade in Michigan, USA (Rosenblum et al. 2013; Schloegel et al. 2012). Although UM-142 may be more closely related to the LFT strain from Brazil (see Rosenblum et al. 2013), it was not possible to make final conclusions regarding its true point of geographic origin (or if it originated from a temperate or tropical region). Therefore, we refer to UM-142 as “Bullfrog” throughout the paper. Genomic data show that both the Tropical and the Bullfrog strains are outside of the GPL clade, and in a clade that is sometimes referred to as “BdBrazil”. The temperature profiles of the regions for the Temperate and Tropical strains likely overlap in the intermediate temperature range for *Bd* growth (11–22 **°**C), but extend beyond that range where the Temperate strain was collected.

### *Bd* growth assays

**Fig. 1:**
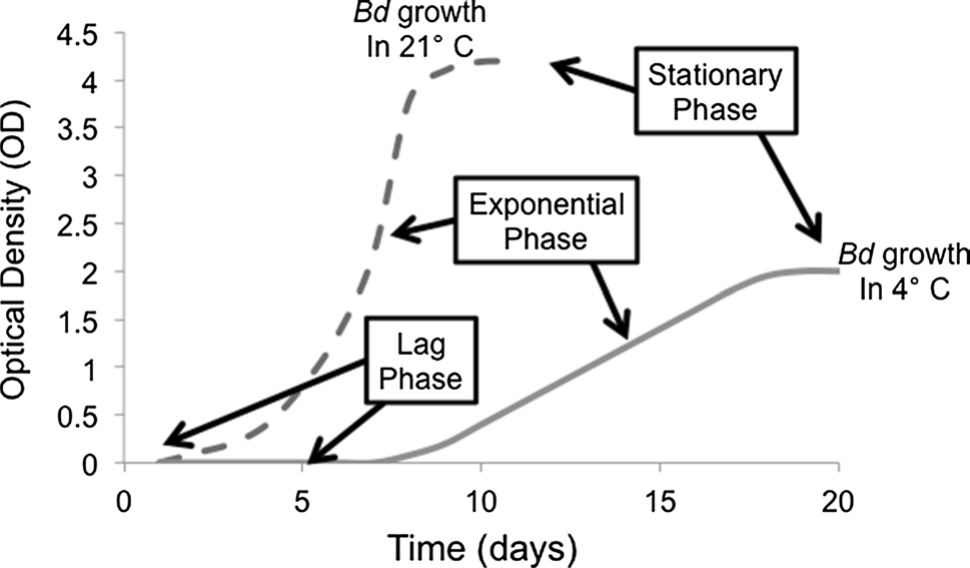
A model of the classical phases of microbial growth (lag phase, exponential phase and stationary phase) for the fungal pathogen *Batrachochytrium dendrobatidis* (*Bd*). Curves represent the growth, measured by optical density (OD), of *Bd* in two temperatures: 21 °C (*dashed line*) and 4 °C (*solid line*)

To measure growth in the three *Bd* strains, we implemented standard microbiological protocols and evaluated microbial growth patterns (Murray et al. 2015). Because we used only zoospores in the plate inoculations, we were able to identify the classical phases of microbial growth, lag phase, exponential phase and stationary phase (see Fig. 1; Murray et al. 2015), by measuring optical density (OD) for multiple successive days. We identified the point of transition from lag phase to exponential phase when there was a detectable increase in OD (Fig. 1). We defined the point of transition from exponential phase to stationary phase (i.e., the “peak” of *Bd* growth) when there were no additional increases in OD (Fig. 1). The goal was to determine how these growth phases differed among the different *Bd* strains across the range of experimental temperature treatments.

To prepare the cultures, we revived aliquots of these *Bd* strains simultaneously, treated them identically, and kept passage histories low. Specifically, we cultured the revived *Bd* strains in TGhL broth (16 g tryptone, 4 g gelatin hydrolysate, 2 g lactose, in 1000 mL distilled water, autoclaved) in 25 cm^2^ flasks. We incubated the flasks at 21 **°**C and passaged *Bd* into fresh TGhL every 7–9 days (Boyle et al. 2003; Voyles 2011). To ensure that only viable zoospores were harvested for the experiment, we centrifuged each strain at 1700*g* for 5 min, 5–7 days prior to the beginning an experiment (Voyles 2011). This step eliminated dead zoospores from all cultures. We then added 2 mL of the supernatant to new 25 cm^2^ flasks with 8 mL of fresh TGhL.

On the first day of the experiment, we filtered 5–7 day old cultures through 0.22 mm filter paper to remove sporangia. For each strain, we counted zoospores using a hemocytometer and diluted the cultures with fresh TGhL to establish a standard zoospore inoculation concentration of 50 ± 3.9 × 10^4^ zoospores per mL. We incubated one aliquot of culture at 40 **°**C for 10 min to generate a heat-killed culture to use as our negative control. We then pipetted the cultures into flat-bottomed, sterile 96-well plates with 50 µL culture broth and 50 µL fresh TGhL per well (*n* = 8 wells per three strains, plus *n* = 8 wells for heat-killed negative control) with 3–5 replicate plates. To prevent desiccation, we pipetted 100 µL TGhL into the perimeter wells of each plate.

We recorded an initial optical density (OD) reading at 490 nm using an E-max precision microplate reader (Molecular Devices, Softmax Pro, Sunnyvale, CA, USA). We recorded OD either once daily (*T* ≥ 12 **°**C) or every other day (*T* ≤ 12 **°**C). Prior to collecting OD measurements, we cleaned the interior surfaces of each plate with a KimWipe to remove any condensation from the plate lid. We continued to record OD daily until we saw evidence of a stationary phase, indicating the end of *Bd* growth.

### Temperature shock experiments

Our first objective was to quantify *Bd* growth in temperatures beyond its previously published *T*_min_ and *T*_max_ (Piotrowski et al. 2004; Stevenson et al. 2013). We were particularly interested to see if *Bd* could survive a temporary freeze treatment or temporary exposure above the highest published temperature for *Bd* (27 **°**C; Piotrowski et al. 2004; Stevenson et al. 2013). For the freeze shock treatments, we inoculated the *Bd* cultures into 96-well plates (as described above) and included wells containing heat-killed *Bd* as a negative control. One plate was incubated in one incubator at 21 **°**C continuously to provide a positive control. We held a second plate in a second incubator (freezer) at −12 **°**C for 24 h, and we then shifted the plate to 21 **°**C for incubation for 7 days. Although a shift of −12–21 **°**C is unlikely to occur in nature, we wanted to move plates from a freezing temperature to the temperature that matched our control treatment, and one that is thought to be an ideal temperature for *Bd* growth (21 **°**C). We verified temperatures in the incubator using a non-contact infrared thermometer (Raytek ST80 Pro-Plus Non-contact thermometer, Santa Cruz, USA).

For the heat shock treatment, we used the same 96-well plate setup with heat-killed *Bd* as a negative control (as described above). We inoculated *Bd* zoospores into three plates, one plate was placed in an incubator at 21 **°**C continuously to provide a positive control and one plate was placed in a second incubator at 28 **°**C for 24 h and then subsequently moved to 21 **°**C, and one plate was maintained in a third incubator at 28 **°**C for the duration of the experiment. For both the freeze and heat shock experiments, we checked all wells for any visual signs of contamination, omitted the results if we observed contamination and then fitted the logistic growth model (described below).

### *Bd* responses to low, high, and intermediate temperatures

In addition to our temperature shock treatments, we used three incubators to conduct growth assays at 2, 3, and 4 **°**C (for low temperature conditions), 26, 27, and 28 **°**C (for high temperature conditions) and at 5, 12, and 21 **°**C (for intermediate conditions). We set up multiple 96-well plates (as described above) including wells with heat-killed *Bd* as a negative control. Following inoculation of *Bd* into the wells, the plates were incubated at each temperature until growth reached a stationary phase (Fig. 1). Because we had access to a limited number of incubators, we could not test the strains in all of the temperatures simultaneously. However, we grouped the experiments by temperature ranges (low, high or intermediate temperatures), used identical protocols for plate setup, and diluted the zoospores to the same inoculating dose (50 ± 3.9 × 10^4^ zoospores per mL) for all experiments.

### Model development

Previous studies have indicated that the *Bd* growth cycle consists of two portions: a motile zoospore phase, and a larger, sessile sporangia state that then produces more zoospores (Longcore et al. 1999; Berger et al. 2004). Zoospores in solution must first settle and they subsequently begin to develop into sporangia, which occurs during the lag phase. These sporangia take time to mature before they produce and release zoospores. If zoospores are monitored and counted independently, many biologically important parameters can be estimated from these data (Voyles et al. 2012, 2014). However, when measuring optical densities (ODs), sporangia and zoospores are counted together. Therefore, we used a simpler model that captures key growth patterns while including important details of the *Bd* life cycle.

Growth curves of various microorganisms in culture, including bacteria and fungi, are typically sigmoidal in nature. Therefore, we chose to model the OD curves obtained above with a modified version of logistic growth. This model incorporates an initial phase of low growth or decay (to capture the possibility that zoospores settle or die, as well as the delay in sporangia maturation) for a fixed period of time, followed by standard logistic growth. The mean growth model for the optical density (D) is given by

$$D = D_{0} {\text{e}}^{ - mt} {I}_{(t\, < \,d)} + \frac{{K\left( {D_{0} {\text{e}}^{ - md} } \right)}}{{(\left( {D_{0} {\text{e}}^{ - md} } \right) + \left( {K - \left( {D_{0} {\text{e}}^{ - md} } \right)} \right)\exp \left( { - r\left( {t - d} \right)} \right)}}I_{{_{(t\, > \,d)} }} .$$

*D*_0_ is the initial optical density, *m* is the decay rate during the initial lag phase, *d* is the length of the initial lag phase, *r* is the per capita growth rate during the logistic phase, and *K* is the carrying capacity (i.e., maximum population size). Thus, we have five primary model parameters to infer from our data and *I* is an indicator that is equal to 1 if the condition is true.

**Fig. 2:**
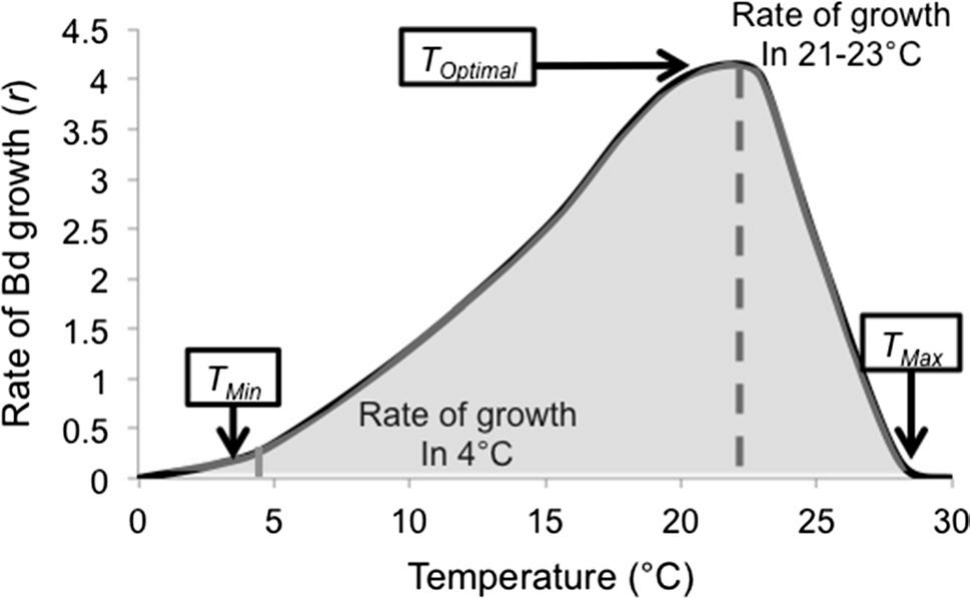
A model of the relationship between temperature and exponential growth rate (*r*) of the lethal fungal pathogen *Batrachochytrium dendrobatidis* (*Bd*). The lines represent an example of *r* for *Bd* in two temperatures: 21 °C (*dashed line*) and 4 °C (*solid line*)

We used a Bayesian inference approach because it allows us to understand the uncertainties in our parameter estimates, even when they are coupled in a more complex non-linear way (Clark 2007). As the OD data cannot be negative, and to capture the increase in variability in the data over time, we used a log-normal distribution where the log-mean is given by Eq. 1. We chose relatively uninformative priors for many of the parameters of the model, specifically growth rate (*r*; see Fig. 2), length of lag phase (*d*) and the decay rate during the lag phase (*m*). We chose the prior for the variance of the observation model 2 to give higher prior weight to models with small variance. To improve convergence, we selected a more narrow range for the prior on the initial density (*D*_0_) and bounded the prior on the carrying capacity (*K*) to disallow values that were above the highest observed OD values across experiments until we saw evidence of the stationary phase.

We conducted the analyses in R (R Core Team 2016) with Markov chain Monte Carlo (MCMC) implemented in rjags/JAGS (Plummer et al. 2003; Plummer 2013). We checked for convergence of the MCMC chains visually and used standard convergence metrics. Model selection was based on the Deviance Information Criterion (DIC) as implemented in rjags/JAGS (Plummer et al. 2003; Plummer 2013). We obtained posterior estimates of all the parameters in Eq. 1, and used these to obtain the posterior distributions of the growth trajectories. We used these parameters to understand the responses of the *Bd* strains during freeze and heat shock experiments and when they were incubated at a range of constant temperatures from 2 to 27 °C.

To assess the overall performances of the *Bd* strains across the different temperatures, we calculated the trajectories from the modified logistic equation based on samples from the posterior distributions of parameters. Using the posterior samples of the growth trajectories, we calculated the area under the curve (AUC) using the auc function in the flux package (Jurasinski et al. 2014) in R (R Core Team 2016).

To characterize the thermal breadth of each strain, we fit a Johnson–Lewin (J–L) curve to subsamples of the AUC and to the growth rate from the modified logistic curve (*r*), across temperatures. The Johnson–Lewin curve is an asymmetric, unimodal curve often used to describe the thermal response of traits (Johnson and Lewin 1946), and is given by

$$\left( {1 + { \exp }\left( {\frac{{T - T_{\text{opt}} }}{T} \times \frac{{E_{D} + E}}{{kT_{\text{opt}} }}} \right)\frac{{E_{D} - E}}{E}} \right)^{ - 1} ,$$

where *c* scales the height of the curve, *T*_opt_ determines the location of the peak, *E*_*D*_ determines the breadth of the curve, *E* determines the shape of the curve, and *k* is the Boltzmann constant.

The thermal breadth is defined as the range of temperatures over which growth of at least 75% of the maximum growth rate occurs. Although basing the breadth on the logistic parameter (*r*) has been done in previous studies (Raffel et al. 2013), *r* will not necessarily capture the overall population growth rate because our models include a delay for lag phase. Therefore, we used an additional measure, the area under the growth curve (AUC), to complement and compare to the thermal breadth measures based on *r*. As with *r*, we defined the thermal breadth for AUC to be the range of temperature across which AUC was at 75% of its maximum.

Where logistic growth occurred (i.e., *r* > 0), we examine alternative definitions of the thermal breadth by examining the fitted J–L curve to see what ranges could be characterized as “low” and “high” logistic growth. Specifically, we defined “low growth rate” as 0.01 > *r* > 0.1. That is, within this range we could be confident of at least some *Bd* growth. We defined “high growth rate” as *r* > 0.1. This approach allowed us to capture more information about the tails of the distribution of growth.

## Results

### Temperature shock experiments

We used a Bayesian analysis of the probability of growth for the freeze (F) and control (C) treatments. Although the posterior samples suggested that the freeze shock treatment reduced the probability that *Bd* will grow, we found that all the *Bd* strains had at least a portion of samples that grew and produced zoospores following a freeze shock of −12 **°**C for 24 h.

**Fig. 3:**
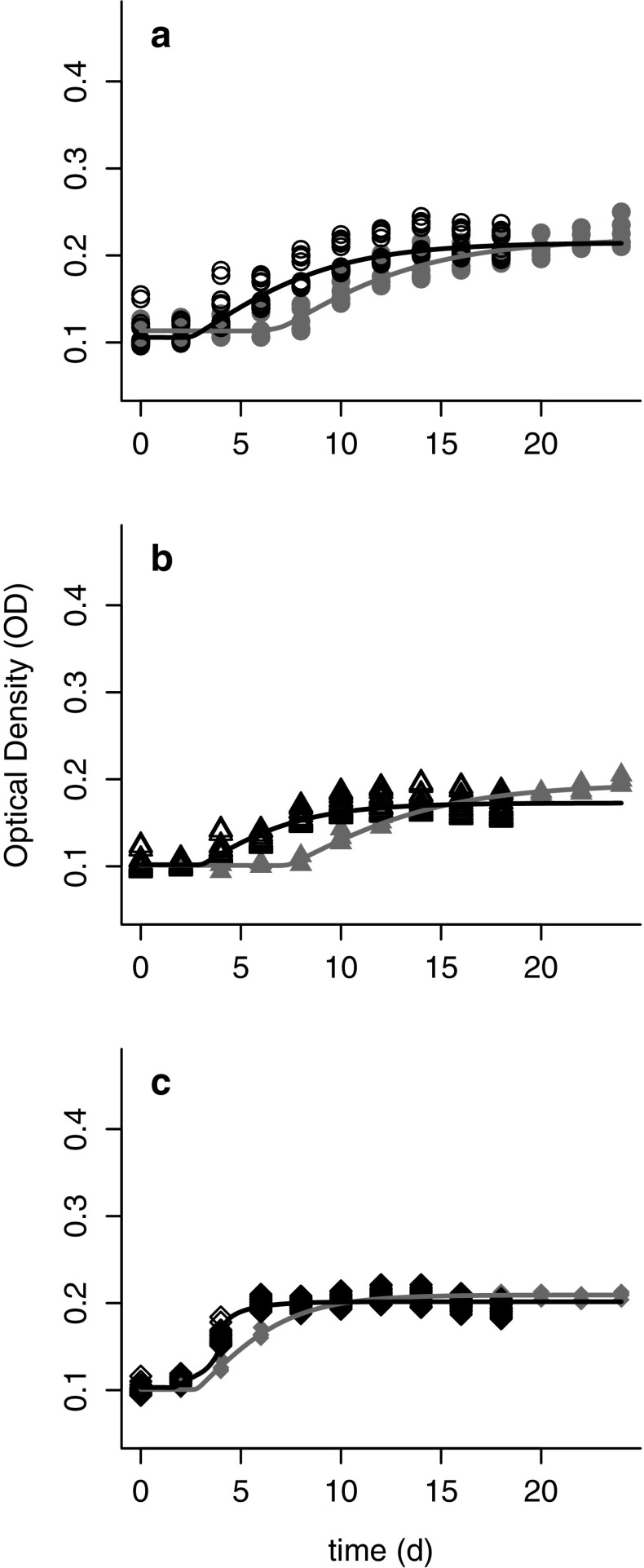
Growth of three strains of *Batrachochytrium dendrobatidis* (*Bd*) representing different phylogenetic clades: CJB5-2 or “Temperate” (**a**), LFT or “Tropical” (**b**) and UM-142 or “Bullfrog” (**c**). Growth was measured by optical density (OD) following either a 12 h freeze shock treatment (*solid shapes*, *solid lines*) or maintained at 21 °C (*open shapes*, *dashed lines*)

Using the fitted logistic model for the samples that exhibited growth, we examined the effects of the freeze shock treatment on the decay rate during the initial lag phase (*m*), the length of the initial lag phase (*d*), the growth rate during the logistic phase (*r*), and the carrying capacity of the logistic phase (*K*). We found that the length of the initial lag phase (*d*) was longer in the freeze shock treatment compared to the control treatment (i.e., the cultures in the freeze shock treatment took longer to initiate growth). However, following the prolonged lag phase in the freeze shock treatment, the *Bd* strains grew well and had higher carrying capacities (*K*) than the cultures in the control treatment (Fig. 3a–c).

**Fig. 4:**
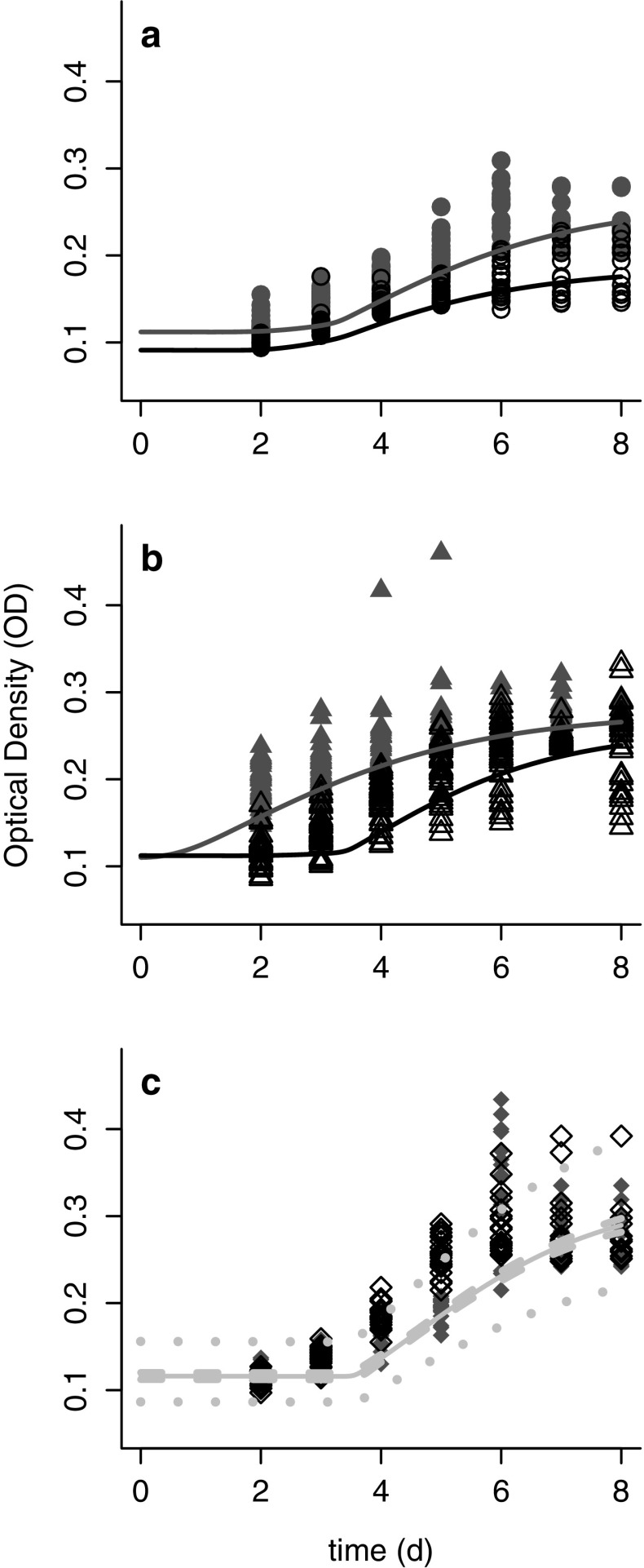
Growth of three strains of *Batrachochytrium dendrobatidis* (*Bd*) representing different phylogenetic clades: CJB5-2 or “Temperate’’ (**a**), LFT or “Tropical” (**b**) and UM-142 or “Bullfrog” (**c**). Growth was measured by optical density (OD) following either a 12 h heat shock treatment at 28 °C (*solid shapes*) or maintained at 21 °C (control; *open shapes*). Note that for the Bullfrog strain the control and heat shock treatments behave in the same way (i.e., a model with both fit as one curve is preferred by DIC)

Following a heat shock of 28 **°**C for 24 h, all *Bd* strains grew and produced zoospores when they were returned to 21 **°**C. However, there were some differences among the cultures exposed to heat shock (HS) and control (C) conditions (Fig. 4a–c). For two of the strains (Temperate and Tropical), the DIC values indicated that a model that uses different parameters in the logistic model better described the data. Specifically, there were differences in the three primary parameters of interest (*r*, *K* and *d*) between treatment and control. For these two strains, the growth rate (*r*) was typically lower after the heat shock, but the carrying capacity (*K*) was higher (Supplementary Materials). Additionally, for the Tropical strain, exposure to high temperature decreased the length of the initial lag phase (*d*), although this effect was not observed in the other *Bd* strains (Supplementary Materials).

### *Bd* responses to a temperature gradient

We evaluated the growth patterns and determined if logistic or constant growth models were more appropriate. The DIC values indicated that, when comparing across most temperatures, the logistic model was preferred over the constant model for all the *Bd* strains. The only exceptions were in the Tropical and Bullfrog strains at 28 **°**C. However, the outcomes were equivocal because one experiment supported constant and the other supported low logistic growth. Therefore, we used the logistic model to evaluate the responses of strain to thermal treatments.

**Fig. 5:**
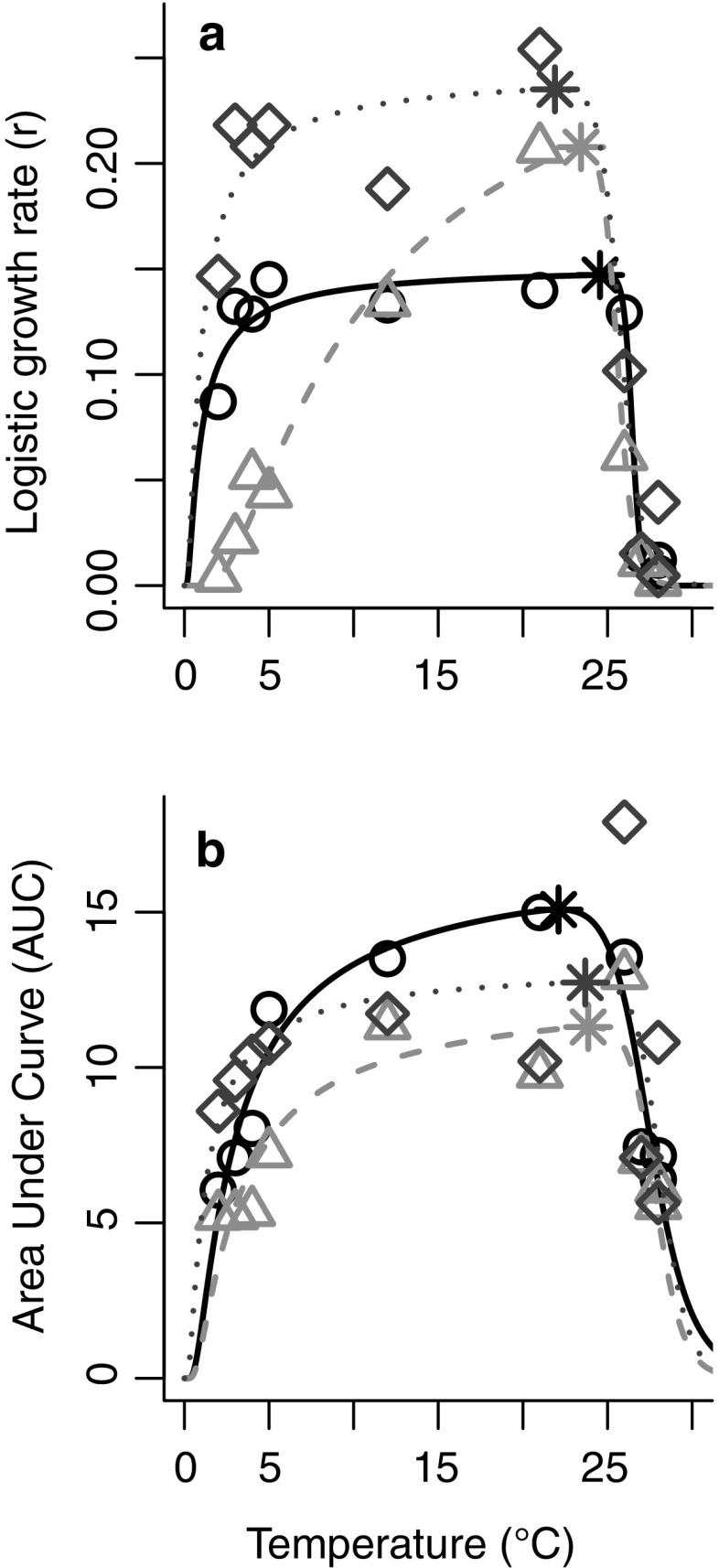
Thermal breadth expressed in terms of **a** Logistic growth rate (*r*) and **b** area under the curve (AUC) for three strains of *Batrachochytrium dendrobatidis* (*Bd*) representing different phylogenetic clades. CJB5-2 or “Temperate” (solid line, open circles), LFT or “Tropical” (*dashed line*, *open triangles*) and UM-142 or “Bullfrog” (*dotted lines* and *open diamonds*) across temperatures (2–28 °C). *Asterisks* (*) indicate the optima for each strain

Across all four methods of measuring thermal breadth, we observed similar patterns for the three *Bd* strains (Table 1). The Tropical strain grew poorly at low temperatures relative to the Temperate and Bullfrog strains (Fig. 5a, b). The Bullfrog strain had the fastest growth rates, including at very low and very high temperatures (Fig. 5a). All three strains exhibit similar upper thermal limits for high growth, at approximately 25–26 **°**C (Fig. 5a, b).

**Table 1:** Thermal breadth of the three strains of *Batrachochytrium dendrobatidis* (*Bd*) isolates calculated 4 ways: *r* > 0.1; *r* > 0.01; width at 75% of the maximum estimated *r* (fit from the Johnson–Lewin curve); width at 75% of the maximum estimated area under the curve (AUC, fit from the Johnson–Lewin curve)

Isolate	Tat *r*_max_	*r* > 0.01	*r* > 0.1	*r* = 0.75 *r*_max_	AUC = 0.75AUC_max_
CJB5-2	24.6	(0.280–27.1)	(1.82–26.3)	(2.35–26.2)	(5.92–26.3)
LFT	23.4	(2.59–27.0)	(7.92–25.6)	(13.0–25.1)	(6.34–27.0)
UM-142	22.1	(0.245–27.9)	(0.876–26.0)	(2.38–25.2)	(2.91–27.4)

Previous studies (Woodhams et al. 2008; Raffel et al. 2013; Piotrowski et al. 2004) suggest that *Bd* should reach peak growth at an intermediate temperature of 21 **°**C. The pattern we observed in the Tropical strain was similar (Fig. 5a), though our results indicate a possibly higher temperature of peak growth (~23 **°**C). However, we found that the growth rates for the Temperate and Bullfrog strains were high and relatively flat across a broad range of temperatures (Fig. 5a), from 2 to 25 **°**C, although the “peaks” occurred at 24.6 **°**C for the Temperate strain and at 22.1 **°**C for the Bullfrog strain (based on the fitted J–L curves, Fig. 5a and Table 1).

## Discussion

The fungal pathogen *Bd* is known to be sensitive to temperature, and it was previously thought that its critical temperature range spanned from 4 to 27 **°**C, with optimal growth between 17 and 21 **°**C (Woodhams et al. 2008; Raffel et al. 2013; Piotrowski et al. 2004; Stevenson et al. 2013). However, these previous studies tested only a single isolate of *Bd*, multiple isolates from within a single *Bd* lineage, or only across a limited temperature range (Woodhams et al. 2008; Raffel et al. 2013; Piotrowski et al. 2004, Stevenson et al. 2013). To build on these previous studies, and to provide a fuller understanding of temperature effects on *Bd*, we selected three *Bd* strains and tested their responses in temperature shock treatments and growth assays across and beyond the previously published tolerance range for *Bd* (Piotrowski et al. 2004).

Our results show that the *Bd* strains exhibited different growth patterns across the putative thermal range of *Bd*. Although the three *Bd* strains generally had similar overall patterns in length of initial lag phase (*d*) and decay rate (*m*), we found that the three strains of *Bd* differed in their growth rates (*r*) and carrying capacities (*K*), which demonstrates diversity among strains in thermal tolerance and performance across a broad temperature spectrum. Additionally, we found that all three isolates continued to grow well in 2–27 **°**C, and following freeze shock and heat shock treatments, which establishes a new thermal sensitivity profile for *Bd*. Lastly, we found that two strains (the Temperate and the Bullfrog strains) differed from the Tropical strain in several intriguing ways.

The responses of the Temperate and Bullfrog strains differed from the Tropical strain in two respects. First, we found that the Temperate and Bullfrog strains had higher logistic growth rates (*r*) and carrying capacities (*K*) at the upper and lower extremities of the temperature range (especially at low temperatures, with high growth at 2–3 **°**C). In contrast, the Tropical strain (collected from Brazil) exhibited relatively lower growth rates and carrying capacities at these same thermal extremes. Second, our AUC analyses indicate that Temperate and Bullfrog strains had a better overall performance compared to the Tropical strain across the entire range of experimental temperatures. Because the point of origin of the Bullfrog isolate is unknown, and because we compared only three isolates, it was not possible to make general conclusions regarding adaptive responses to thermal conditions for all of our *Bd* strains. However, it is intriguing that the Bullfrog isolate, which is more closely related to the Tropical isolate (based on genomic data; see Rosenblum et al. 2013), exhibited striking differences from the Tropical isolate in overall performance. Additional research that includes multiple representative isolates from different thermal regions will be necessary to confirm these findings, and to better understand if variation in responses to temperature are genetically determined, or adaptive in a particular thermal environment.

While the idea of *Bd* adaptation to thermal conditions requires further investigation, our results indicate that there is variation among *Bd* strains in thermal tolerance and in overall performance, which are both important findings for different reasons. Because we know that the risk of mortality is proportional to the *Bd* load on an amphibian (Raffel et al. 2013), a *Bd* strain with better overall performance across a broad temperature breadth may be more threatening for amphibian hosts that utilize heterogeneous thermal environments. However, for amphibian hosts that occur in environments where temperatures reach the extremes of the thermal spectrum for *Bd*, a strain that can grow well in such conditions will likely also be problematic [e.g., if *Bd* survives overwintering events (e.g., Knapp et al. 2011) or persists despite host behavioral regulation of body temperatures (Rowley and Alford 2013)]. While the magnitude of these temperature-related effects will be specific to the particular environment and mediated by host biology (including life history, behavior and inherent *Bd* resistance/tolerance), the temperature sensitivity of *Bd* local strain(s) could considerably influence disease development and the propensity of *Bd* to cause devastating outbreaks.

Diversity in thermal tolerance among *Bd* isolates may help explain why amphibians have experienced severe outbreaks in regions where temperature conditions are considered sub-optimal for *Bd* (e.g., Knapp et al. 2011). For example, in the Sierra Nevada Mountains of California, the mountain yellow-legged frog (*Rana muscosa* and *R. sierra* species complex; Vredenburg et al. 2010) continues to experience disease-related declines despite the fact that these host species spend considerable time in temperatures that are lower than the thermal optimum of *Bd* (Knapp et al. 2011). Indeed, these amphibian hosts experience a wide range of temperatures (<0–30 **°**C) that can fluctuate dramatically on a daily and seasonal basis (Knapp et al. 2011). Our results suggest that the thermal profile for the Temperate strain (CJB5-2), which was collected from *R. muscosa* in this region, may contribute to severity of the chytridiomycosis outbreaks and declines in this species. The Temperate strain exhibited a better overall performance across a wider thermal breadth, had high growth rates at low temperatures, and was able to grow following a temporary freeze without cryoprotectant. However, to fully explain the severity of chytridiomycosis in these species, we need a better understanding of the diversity of strains of *Bd* among and within host populations (Byrne et al. 2016), the responses to a variety of stable and fluctuating thermal conditions (Raffel et al. 2013), and the species-specific host responses to temperature conditions that are important for disease development.

Beyond the implications for amphibians, it is critical to understand fungal pathogen responses to thermal conditions for a variety of reasons. First, fungi are generally known for readily adapting to their temperature conditions, but the mechanisms that dictate fungal thermal tolerance and temperature-dependent pathogenesis are not well understood (Feller and Gerday 2003). Therefore, investigations that aim to resolve these mechanisms are of central importance for multiple medically important fungal diseases (e.g., Candida; Antley and Hazen 1988). Second, fungal pathogens have been implicated in many novel fungal diseases in wildlife, including White nose syndrome in bats, colony collapse disorder in bees, and a facial fungal disease in snakes (Voyles et al. 2015; Langwig et al. 2015). Together with chytridiomycosis, these emerging fungal diseases have caused—and continue to cause—dramatic losses of biodiversity. Researchers and wildlife managers are just beginning to confront these disease threats (Voyles et al. 2015; Langwig et al. 2015) and will greatly benefit from understanding the environmental conditions that allow these pathogens to emerge, spread, and cause high levels of host mortality. Third, rapidly changing environments are predicted to shift fungal disease dynamics, but understanding complexities of fungal responses to temperature will be important for anticipating the disease impacts (Raffel et al. 2013; Rohr et al. 2013). For example, it has been suggested that mismatches in thermal tolerances of *Bd* and amphibian hosts could drive chytridiomycosis outbreaks (Nowakowski et al. 2016; Cohen et al. 2017). Therefore, a shifting climate, or an introduction of a *Bd* strain to a thermal environment that it was not adapted to, could dramatically affect the propensity of *Bd* to cause an outbreak (Nowakowski et al. 2016; Cohen et al. 2017).

We suggest that investigations that focus on the responses of hosts and pathogens to temperature will help advance the rapidly growing field of disease ecology. In particular, we suggest that pathogen responses to low temperatures may be currently understudied, and could be key to understanding what is driving disease dynamics in recent catastrophic fungal pandemics. The critical thermal minima of microbes have traditionally been underestimated, probably due, at least in part, to the lack of refrigerating incubators (Morita 1975; Rohr et al. 2013). It is also possible that we have yet to fully appreciate the ubiquity of psychrophilic microbes (Stokes and Redmond 1966), particularly psychrophilic and psychro-tolerant fungi. Given that many devastating fungal pandemics are occurring in ectotherms (e.g., amphibian chytridiomycosis, snake fungal facial disease; Voyles et al. 2015; Langwig et al. 2015) or in animals that undergo torpor events (e.g., White nose syndrome in bats), we suggest that an integration of thermal biology and disease ecology is timely and may prove critical for developing appropriate conservation strategies for infectious diseases in wildlife.

## Electronic supplementary material

Below is the link to the electronic supplementary material.

Supplementary material 1 (PDF 123 kb)
