# Supplementary material for: Diversity in growth patterns among strains of the lethal fungal pathogen Batrachochytrium dendrobatidis across extended thermal optima
Source: Oecologia. 2017 Apr 19;184(2):363–73. doi: 10.1007/s00442-017-3866-8 (PMC5487841; doi:10.1007/s00442-017-3866-8)
Supplement: Supplementary file 1 — Supplementary material 1 (PDF 123 kb) [file 442_2017_3866_MOESM1_ESM.pdf]

Supplementary Material to: Diversity in growth patterns among isolates of the lethal fungal pathogen *Batrachochytrium dendrobatidis* across extended thermal optima

[a]*Department of Biology, University of Nevada- Reno, Reno, Nevada 87801, USA*
[b]*Department of Integrative Biology, University of South Florida, Tampa, FL 33620, USA*
[c]*Department of Statistics, Virginia Polytechnic Institute and State University, Blacksburg, VA, 24061, USA*
[d]*Department of Biology, University of Washington, Seattle, WA 98195, USA*
[e]*New Mexico Tech, Department of Biology, 801 Leroy Place, Socorro, NM 87801, USA*
[f]*Department of Environmental Science, Policy and Management, University of California- Berkeley, Berkeley, California 94720-3144, USA*
[g]*corresponding author: jvoyles@unr.edu, Phone: 720-883-2341*

Abstract

This document contains supplementary methods, results, and figures, as described in the main text of "Diversity in growth patterns among isolates of the lethal fungal pathogen *Batrachochytrium dendrobatidis* across extended thermal optima".

1. Freeze Shock Experiment

	r	K	d	m
F	0.248 (0.138 - 0.363)	0.221 (0.205 - 0.239)	6.68 (5.25 - 7.76)	8e-04 (7.4e-08 - 0.00239)
C	0.278 (0.227 - 0.327)	0.215 (0.208 - 0.222)	2.54 (2.04 - 3.03)	0.001 (2.79e-09 - 0.00301)

Table 1: CJB5-2 isolate

	r	K	d	m
F	0.243 (0.185 - 0.3)	0.195 (0.186 - 0.204)	7.38 (6.83 - 7.89)	0.000885 (6.81e-08 - 0.00259)
C	0.343 (0.298 - 0.391)	0.173 (0.17 - 0.175)	3.06 (2.76 - 3.35)	0.001 (1.98e-07 - 0.003)

Table 2: LFT isolate

	r	K	d	m
F	0.43 (0.377 - 0.48)	0.209 (0.207 - 0.212)	2.8 (2.53 - 3.05)	0.000814 (1.67e-08 - 0.00241)
C	3.58 (0.566 - 13.4)	0.202 (0.199 - 0.204)	2.79 (1.61 - 3.93)	0.000684 (8.68e-09 - 0.0022)

Table 3: UM-142 isolate

2. Heat Shock Experiment

	r	K	d	m
H	0.536 (0.402 - 0.688)	0.262 (0.242 - 0.283)	2.9 (1.61 - 3.55)	0.001 (5.04e-09 - 0.00304)
C	0.573 (0.385 - 0.849)	0.187 (0.173 - 0.202)	2.72 (1.69 - 3.57)	0.001 (1.58e-07 - 0.00299)

Table 4: CJB isolate

	r	K	d	m
H	0.492 (0.354 - 0.637)	0.277 (0.265 - 0.291)	0.573 (0.000107 - 1.23)	0.001 (1.39e-07 - 0.00303)
C	0.629 (0.438 - 0.809)	0.257 (0.24 - 0.278)	3.27 (2.17 - 3.73)	0.000995 (5.3e-08 - 0.00299)

Table 5: LFT isolate

	r	K	d	m
H	0.463 (0.276 - 0.606)	0.377 (0.301 - 0.483)	3.42 (2.52 - 4.61)	0.00102 (3.63e-08 - 0.00309)
C	0.803 (0.688 - 0.922)	0.305 (0.291 - 0.32)	3.62 (3.48 - 3.76)	0.001 (3.31e-08 - 0.00299)

Table 6: UM isolate

3. Temperature Gradient Experiment

	r	K	d	m
2C	0.0871 (0.0792 - 0.0947)	0.367 (0.35 - 0.386)	37.8 (37.2 - 38.4)	3.34e-05 (1.42e-09 - 9.85e-05)
3C	0.132 (0.123 - 0.141)	0.322 (0.316 - 0.328)	31.9 (31.4 - 32.3)	6.25e-05 (8.98e-10 - 0.000183)
4C	0.129 (0.118 - 0.144)	0.298 (0.293 - 0.303)	25.3 (24.7 - 26.5)	7.53e-05 (2.51e-09 - 0.000223)
5C	0.145 (0.136 - 0.155)	0.453 (0.441 - 0.465)	19 (18.6 - 19.4)	0.000115 (4.46e-09 - 0.000337)
12C	0.133 (0.125 - 0.142)	0.346 (0.339 - 0.354)	3.12 (2.63 - 3.56)	0.0012 (7.88e-08 - 0.00357)
21C	0.14 (0.13 - 0.15)	0.374 (0.357 - 0.393)	1.18 (1 - 1.44)	0.00106 (5.63e-08 - 0.00313)
26C	0.129 (0.118 - 0.141)	0.336 (0.318 - 0.355)	1.09 (1 - 1.26)	0.000982 (2.08e-09 - 0.00295)
27C	0.0148 (0.012 - 0.0182)	0.479 (0.323 - 0.6)	1.13 (1 - 1.4)	0.000717 (1.23e-07 - 0.00213)
28C	0.00597 (0.00378 - 0.00827)	0.455 (0.276 - 0.6)	1.28 (1 - 1.87)	0.000652 (2.64e-08 - 0.00194)
28.mC	0.0121 (0.00944 - 0.0151)	0.491 (0.338 - 0.6)	1.67 (1 - 2.54)	0.000934 (6.73e-08 - 0.00282)

Table 7: CJB5-2 isolate

	r	K	d	m
2C	0.0042 (0.00305 - 0.00649)	0.361 (0.173 - 0.585)	9.41 (6.05 - 12.9)	0.00348 (0.000681 - 0.00698)
3C	0.0211 (0.00645 - 0.0309)	0.139 (0.122 - 0.188)	14.5 (9.15 - 18)	0.000273 (8.28e-09 - 0.000826)
4C	0.0525 (0.0416 - 0.064)	0.123 (0.121 - 0.125)	19.4 (17.3 - 21.4)	0.000126 (6.14e-09 - 0.000382)
5C	0.0437 (0.0359 - 0.0518)	0.257 (0.23 - 0.288)	8.73 (7.12 - 10.4)	0.00613 (3.57e-07 - 0.0122)
12C	0.134 (0.128 - 0.141)	0.282 (0.278 - 0.286)	3.37 (3.03 - 3.71)	0.00389 (8.83e-09 - 0.0107)
21C	0.207 (0.198 - 0.217)	0.215 (0.213 - 0.218)	1.62 (1.44 - 1.8)	0.00498 (1.45e-07 - 0.0139)
26C	0.0623 (0.0504 - 0.0762)	0.444 (0.33 - 0.582)	1.09 (1 - 1.28)	0.000965 (3.07e-08 - 0.00289)
27C	0.0113 (0.00878 - 0.014)	0.49 (0.337 - 0.6)	1.26 (1 - 1.78)	0.000765 (2.33e-08 - 0.00229)
28C	0.00189 (3.87e-08 - 0.00395)	0.387 (0.149 - 0.6)	4.11 (1 - 16.7)	0.000544 (6.46e-09 - 0.00175)
28.mC	0.00277 (0.000487 - 0.00586)	0.0199 (0.01 - 0.0402)	19.6 (12.3 - 24.7)	0.000581 (1.85e-08 - 0.00138)

Table 8: LFT isolate

	r	K	d	m
2C	0.147 (0.142 - 0.152)	0.359 (0.355 - 0.362)	25.2 (25 - 25.5)	4.26e-05 (1.7e-09 - 0.000128)
3C	0.211 (0.187 - 0.231)	0.325 (0.323 - 0.328)	22.3 (21.4 - 23)	4.73e-05 (2.24e-09 - 0.000142)
4C	0.215 (0.199 - 0.253)	0.315 (0.312 - 0.317)	17.7 (17.3 - 18.9)	7.34e-05 (6.75e-09 - 0.000224)
5C	0.218 (0.207 - 0.231)	0.343 (0.338 - 0.348)	18.8 (18.5 - 19.1)	0.000215 (8.61e-09 - 0.000627)
12C	0.188 (0.174 - 0.203)	0.281 (0.277 - 0.286)	4.52 (4.12 - 4.91)	0.00232 (2.34e-08 - 0.00656)
21C	0.254 (0.243 - 0.265)	0.222 (0.22 - 0.224)	1.83 (1.69 - 1.98)	0.00278 (1.44e-07 - 0.00838)
26C	0.103 (0.0927 - 0.115)	0.547 (0.481 - 0.6)	1.78 (1 - 2.56)	0.000994 (1.74e-08 - 0.00296)
27C	0.0153 (0.0129 - 0.0181)	0.499 (0.354 - 0.6)	1.21 (1 - 1.64)	0.000801 (4.5e-08 - 0.00239)
28C	0.00473 (0.00175 - 0.00841)	0.0175 (0.01 - 0.0211)	19.7 (19.1 - 20.4)	0.000109 (3.14e-09 - 0.000327)
28.mC	0.0397 (0.036 - 0.0439)	0.556 (0.479 - 0.6)	2.73 (1.42 - 4.07)	0.00102 (1.07e-07 - 0.00306)

Table 9: UM-142 isolate
